# Treatment in a Case of an Overdose of Chloroform

**Published:** 1859-06

**Authors:** 


### TREATMENT IN CASE OF AN OVER-DOSE OF CHLOROFORM.

Dr. Erichson in his excellent work on Surgery says: "the treatment of an over-dose of chloroform is conducted on two principles: 1st, the establishment of respiration, either natural or artificial, so as to empty the lungs of the vapor contained in the air-cells, and to aid the oxygenation of the blood; and 2d, the stimulation of the heart's action, and the maintenance of the circulation.

The first principle of treatment—that of reëstablishing respiration, is most serviceable in the asphyxial form; the other, that of stimulating the heart, when the syncopal symptoms are present. But in all cases they may most advantageously be employed in combination. The treatment to be adopted on the occurrence of dangerous symptoms, or apparent death from chloroform, is as follows:

1. The administration of the vapor must at once be discontinued.

2. The tongue should be seized with the fingers, with a hook or forceps, and drawn out of the mouth: and the larynx pushed up so that the glottis may be opened.

3. Fresh air should be admitted around the patient by opening doors and windows, and by preventing bystanders or spectators from crowding around.

4. All constriction should be removed from the patient's throat and chest, and these parts freely exposed.

5. Artificial respiration must *at once* and without delay be set up, while these other means are being carried out, either by the surgeon applying his mouth to the patient's lips and thus breathing into the chest; or, what is preferable, by the alternate and steady compression and relaxation of the patient's chest.

6. Electricity should be applied freely over the heart and diaphragm through the spine, by means of the Electro-Magnetic or other convenient apparatus.

7. Friction may be employed to the extremities; a little brandy rubbed inside of the mouth; and cold water dashed on the face, as accessory means.

